# Supplementary figures and images for: A nowcasting framework for correcting for reporting delays in malaria surveillance
Source: PLoS Comput Biol. 2021 Nov 16;17(11):e1009570. doi: 10.1371/journal.pcbi.1009570 (PMC8659367; doi:10.1371/journal.pcbi.1009570)

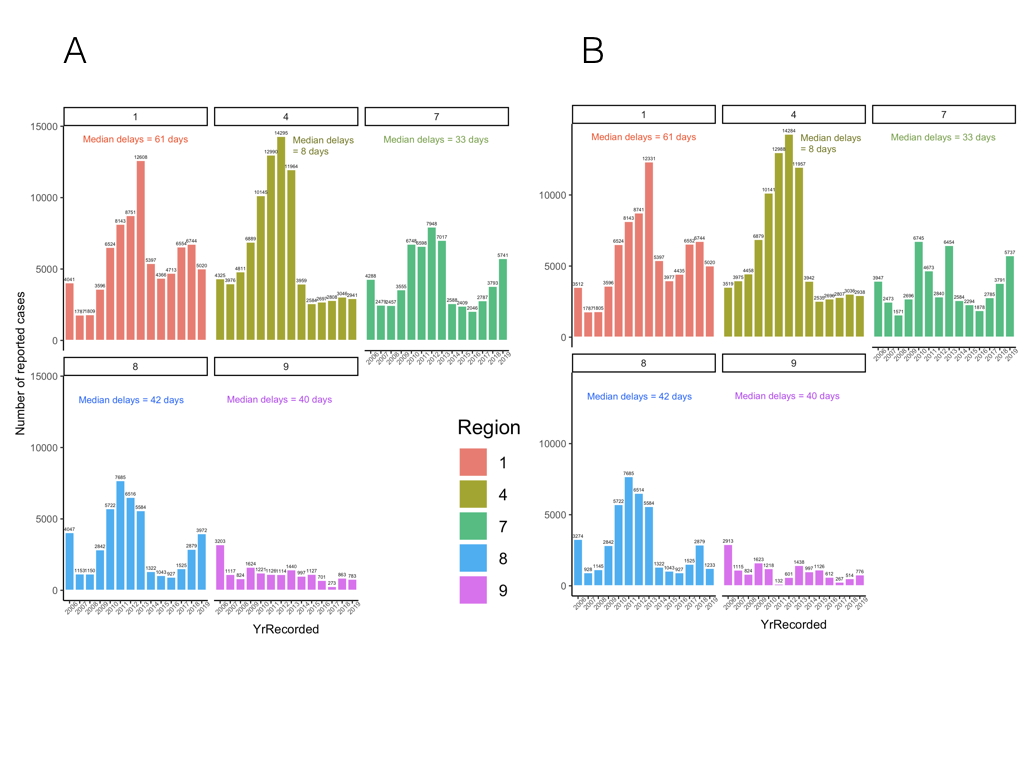

Supplement: S1 Fig — (A) For all patients (B) Excluding patients with implausible recorded date ranges, i.e. were known to be cases at local hospitals after they were known to be cases at the central office in Georgetown). Plot labels indicate the median of delays for all cases from 2006–2019 for each region. Note the relatively low case counts in region 9 from 2016 onwards, particularly after excluding cases with implausible date ranges, lending to small cell counts when parsing the number of cases occurring in a given month known by the end of each subsequent month and the consequent failure for the region 9 models to converge. (TIFF) [file pcbi.1009570.s001.tiff]

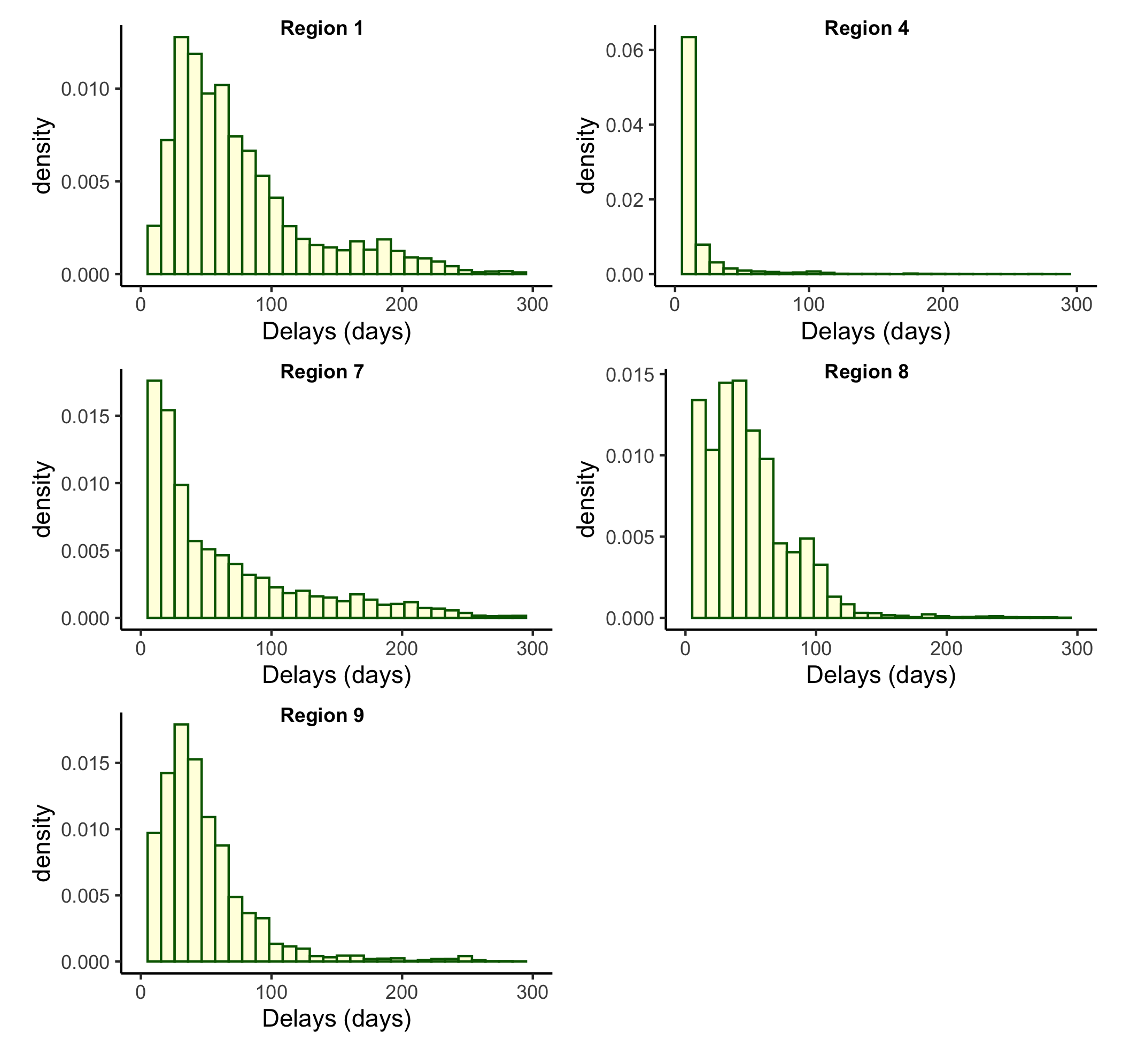

Supplement: S2 Fig — (TIFF) [file pcbi.1009570.s002.tiff]

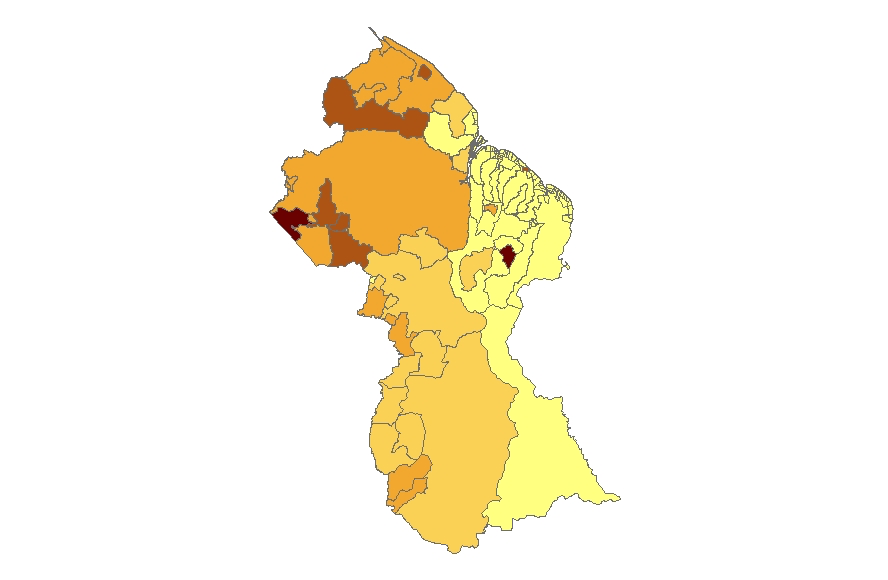

Supplement: S3 Fig — Base map sourced from DIVA-GIS. (TIFF) [file pcbi.1009570.s003.tiff]

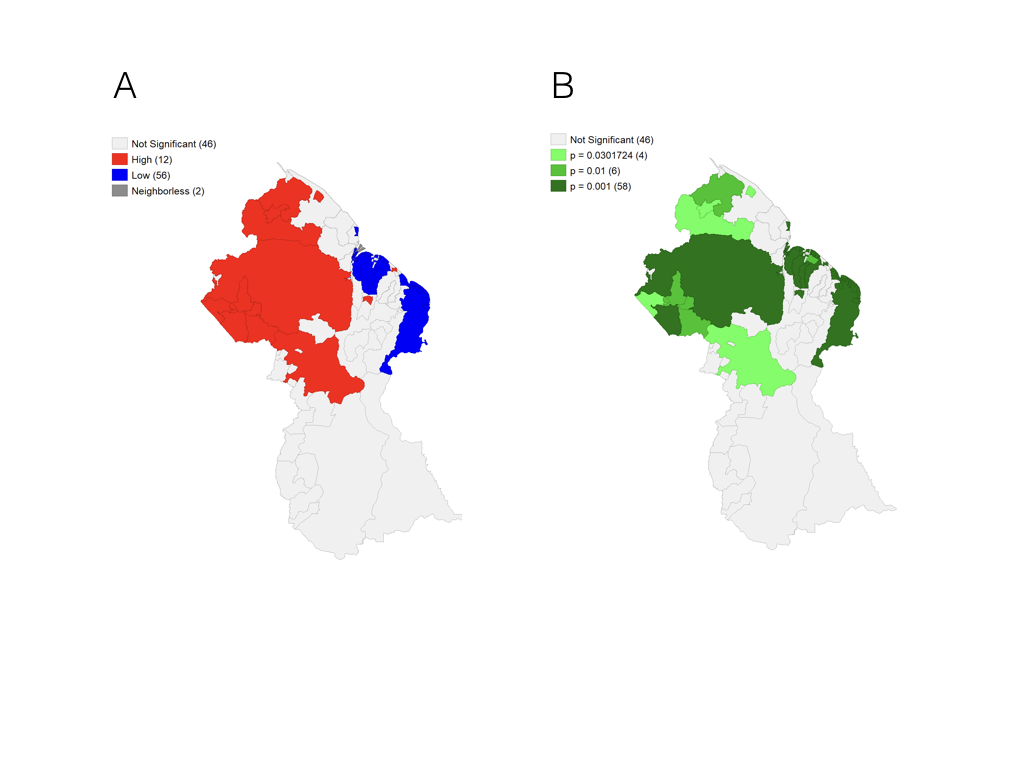

Supplement: S4 Fig — (A) Map of G* statistic (B) FDR-adjusted significance map. Base map sourced from DIVA-GIS. (TIFF) [file pcbi.1009570.s004.tiff]

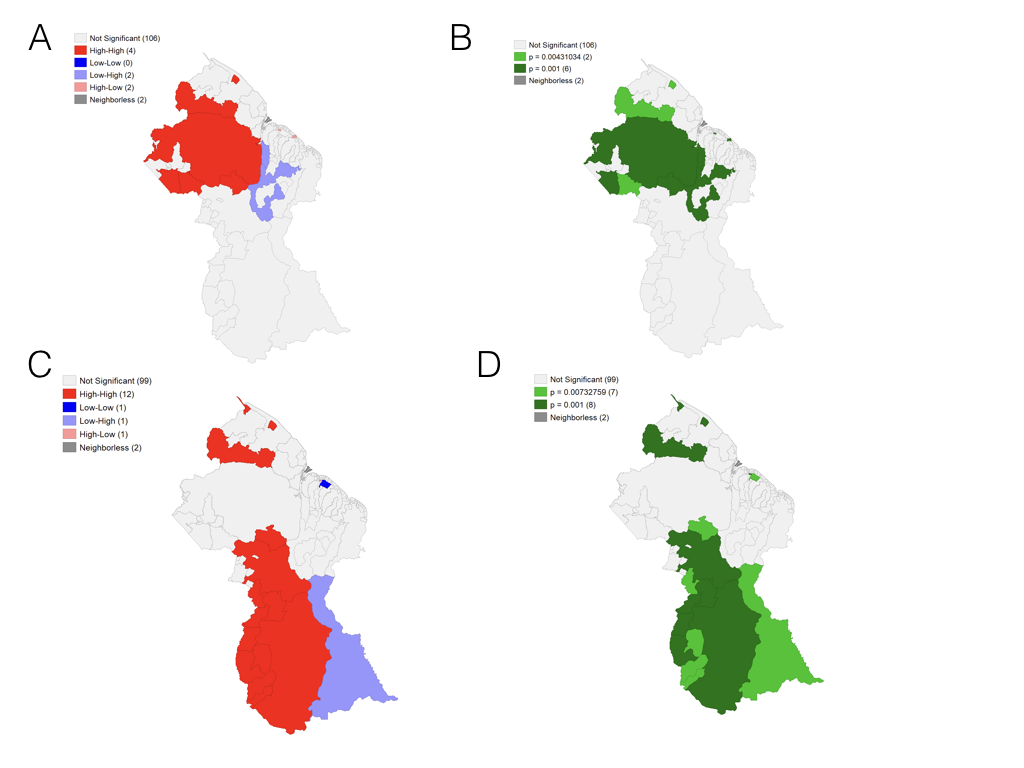

Supplement: S5 Fig — (A) Aggregated median delays and density of mines (B) FDR-adjusted significance map. (C) Aggregated median delays and density of Amerindian settlements (D) FDR-adjusted significance map. Source for base map: https://www.diva-gis.org/datadown. (TIFF) [file pcbi.1009570.s005.tiff]

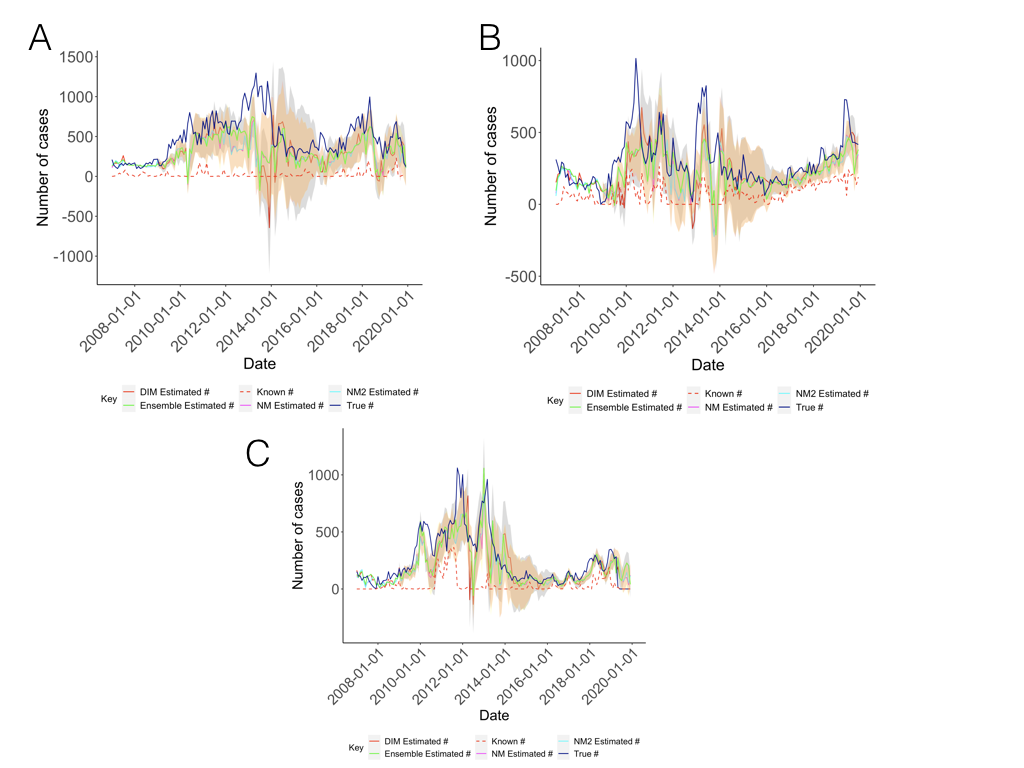

Supplement: S6 Fig — Results from all models: DIM (red, solid), NM1 (magenta), NM2 (cyan), and the ensemble model (green). (A) Region 1 (B) Region 7 (C) Region 8. As before, dashed red lines indicate the number of cases known by the end of the month and solid blue lines indicate the true number of eventually reported cases. Grey bands capture the moving 95% confidence intervals for each model. (TIFF) [file pcbi.1009570.s006.tiff]

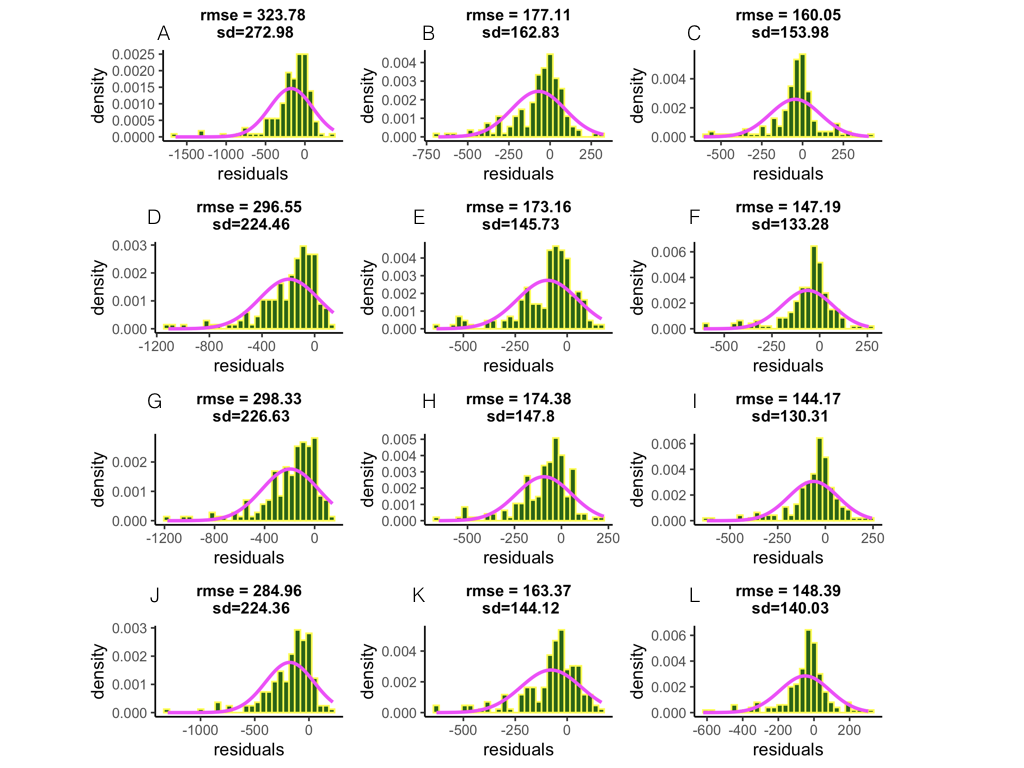

Supplement: S7 Fig — Histogram of monthly residuals (model predicted- eventually reported cases) from January 2007—December 2019 and overlaid Gaussian density curve for (A) Region 1 DIM (B) Region 7 DIM (C) Region 8 DIM (D) Region 1 NM1 (E) Region 7 NM1, (F) Region 8 NM1 (G) Region 1 NM2 (H) Region 7 NM2 (I) Region 8 NM2. (J) Region 1 Ensemble (K) Region 7 Ensemble (L) Region 8 Ensemble. All panels report the rmse of the corresponding model predictions and standard deviation of the corresponding residuals. Histogram + Gaussian density curve for region 4 DIM (rmse = 122.38 and sd = 122.00). (follows the format provided in Poirier et al [35]. (TIFF) [file pcbi.1009570.s007.tiff]

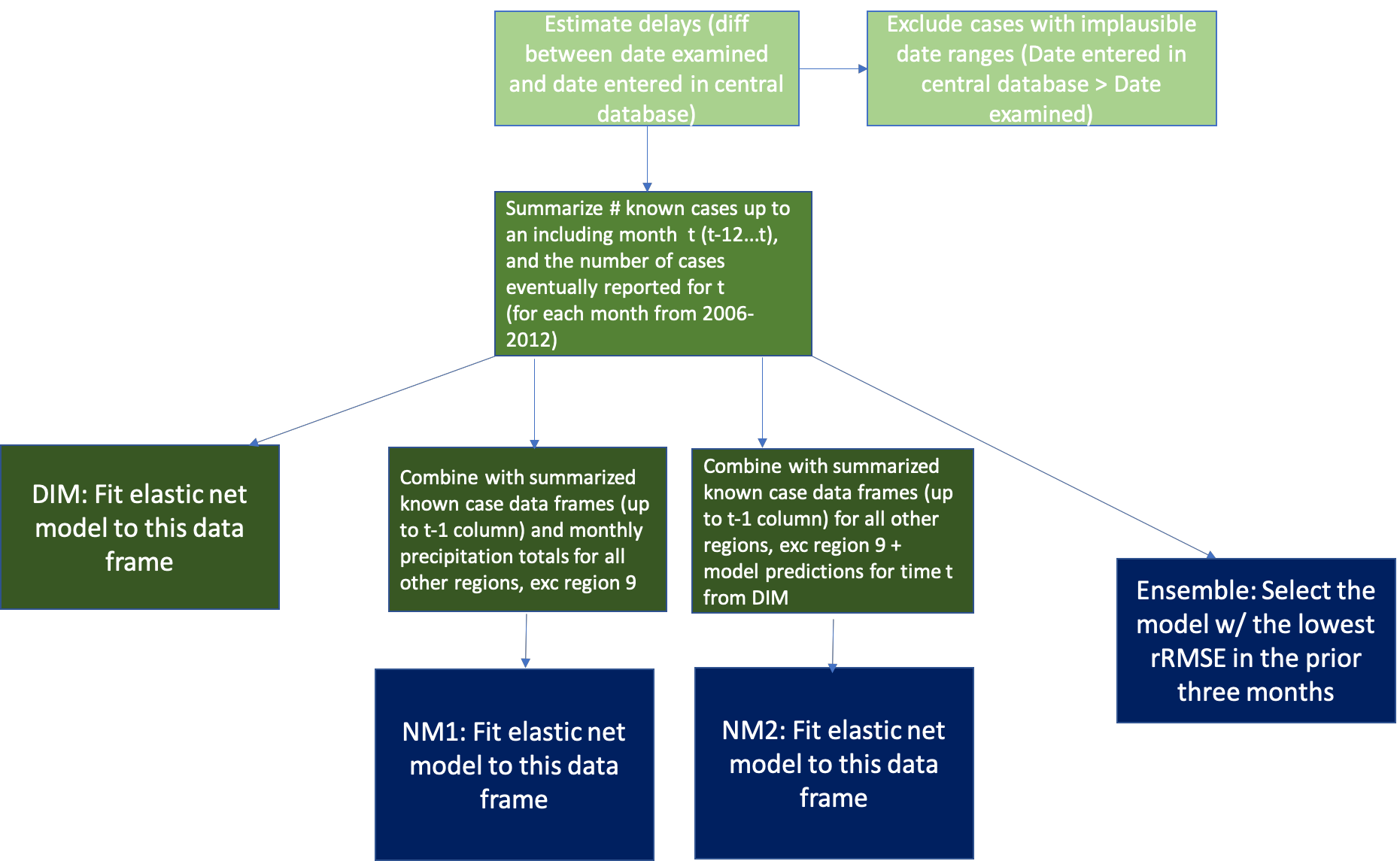

Supplement: S1 Diagram — (TIFF) [file pcbi.1009570.s009.tiff]

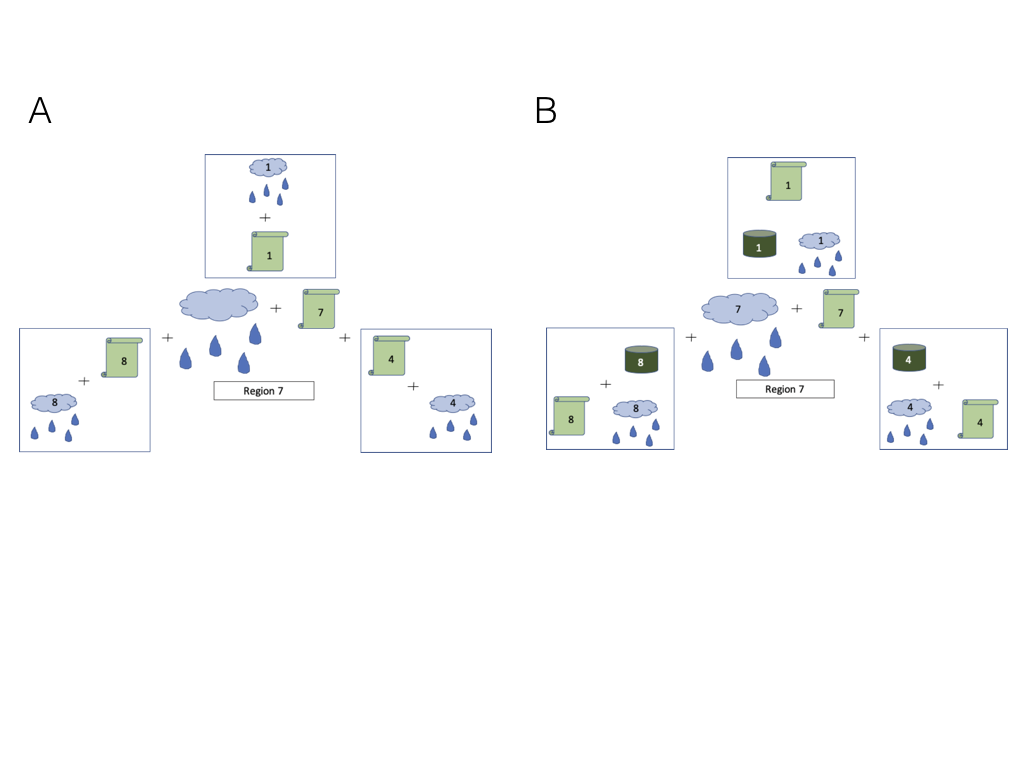

Supplement: S2 Diagram — (a) Diagram detailing each of the components of the first network model, i.e. monthly known case counts up to t-1 for all regions j≠i and for known case counts up to t for region i (scroll, shaded in light green) and monthly precipitation levels for all regions j in month t (cloud with rain, shaded in light blue) (b) Diagram detailing each of the components of the second network model, i.e. monthly known case counts up to t-1 for all regions j≠i and for known case counts up to t for region i (scroll, shaded in light green), DIM predicted case counts for month t for all regions j≠i (cylinder, shaded in dark green), and monthly precipitation levels for all regions j in month t (cloud with rain, shaded in light blue). For both a and b, we use region 7 as the example region of interest for which we are producing the network model estimates. (TIFF) [file pcbi.1009570.s010.tiff]
