# Supplementary material for: A nowcasting framework for correcting for reporting delays in malaria surveillance
Source: PLoS Comput Biol. 2021 Nov 16;17(11):e1009570. doi: 10.1371/journal.pcbi.1009570 (PMC8659367; doi:10.1371/journal.pcbi.1009570)
Supplement: S1 Table — Note: for region 4, rRMSE only reported for the data imputation model, given that no network models were run for this region). The final row reports the best performing model* for each region, excluding the ensemble, which corresponds to the model resulting in the lowest rRMSE. (DOCX) [file pcbi.1009570.s008.docx]

|  | region 1 | region 4 | region 7 | region 8 |
| --- | --- | --- | --- | --- |
| DIM | 0.2703 | 0.0781 | 0.1744 | 0.1508 |
| NM 1 | 0.2475 | NA | 0.1706 | 0.1387 |
| NM 2 | 0.2490 | NA | 0.1718 | 0.1359 |
| Ensemble | 0.2379 | NA | 0.1610 | 0.1399 |
| Best performing model* | **NM1** | **DIM** | **NM1** | **NM2** |

**S1** **Table. Table of relative root mean squared errors (rRMSE) generated from data imputation models and network models for each region**. Note: for region 4, rRMSE only reported for the data imputation model, given that no network models were run for this region). The final row reports the best performing model* for each region, excluding the ensemble, which corresponds to the model resulting in the lowest rRMSE.
