# Supplementary material for: A nowcasting framework for correcting for reporting delays in malaria surveillance
Source: PLoS Comput Biol. 2021 Nov 16;17(11):e1009570. doi: 10.1371/journal.pcbi.1009570 (PMC8659367; doi:10.1371/journal.pcbi.1009570)
Supplement: Attachment — Submitted filename: Review_Malaria.pdf [file pcbi.1009570.s011.pdf]

Summary of review:

The article is a clearly written and distinct contribution to the literature on correcting reporting delays and nowcasting diseases, which is currently receiving considerable attention due to the ongoing pandemic.

The authors have done a great job of motivating the problem of reporting delays through their compelling application to malaria in Guyana. I particularly appreciate the descriptions of specific causes of reporting delays in the introduction.

The authors present two classes of models and their application to the malaria data. Leveraging these models and a more descriptive analysis, the authors discuss new insights into the data, including the spatial distribution of reporting delays, and demonstrate a level of effectiveness as a tool for nowcasting case counts.

However, I do believe there are some weaknesses in the article which should be addressed prior to publication.

Major comments:

1. The authors make a potentially misleading claim about other approaches, specifically those designed in the Bayesian framework.

"One limitation of the Bayesian methods is that they do not focus on providing more interpretable measures to guide actionable surveillance efforts, such as direct point estimates for predicted case counts."

The cited Bayesian approaches (e.g. McGough et al. and Bastos et al.) are capable of producing *both* point estimates (e.g. posterior median, posterior mode) and measures of uncertainty (e.g. 95% prediction intervals) for the total number of cases. I believe these point estimates are no less valid or direct than point estimates derived by minimising a mean squared error.

2. The authors do not address the lack of measures of uncertainty in their approach, particularly relating to the predicted case counts.

Given that all nowcasting predictions from any model will be in some sense "wrong", it is my opinion that measures of uncertainty are important for disease surveillance applications, both to quantify how wrong the predictions might be and also, for example, to inform decision-makers about possible scenarios other than the most likely scenario. Measures of uncertainty are apparently lacking from the authors' approach, though they don't acknowledge this as a potential drawback.

If used in an operational setting over a period of time, one would begin to collect information about the (out-of-sample) prediction errors of nowcasts from these models. Could you derive approximate measures of prediction uncertainty from the distribution of these errors? For example (I am not specifically recommending this solution), in unpublished works, I have previously derived crude intervals from twice the standard deviation (without any statistical justification) of the last N errors, so that uncertainty is measured in a moving window.

3. The authors have missed out a growing literature of Bayesian approaches which combine a model for the total case counts occurring in each time period with a conditional model for the reporting delay. These hierarchical approaches result in a predictive distribution for the total case counts, directly informed by all available partial observations of the number of cases and previously observed case counts. In my opinion, these approaches, particularly those based on the Generalized-Dirichlet-Multinomial framework, represent the current “gold standard” of nowcasting infectious disease. Therefore, I would like to see these discussed alongside the other existing approaches.

Originating article for the GDM framework:

[1] https://doi.org/10.1111/biom.13188

Applications to COVID-19:

[2] https://arxiv.org/pdf/2102.04544.pdf

[3] https://doi.org/10.1101/2020.09.15.20194209

[4] https://arxiv.org/abs/1912.05965

4. While the proposed models are apparently novel, without any quantitative comparisons with other approaches it is difficult to place them in the context of existing approaches and therefore determine the value of their contribution to the problem of nowcasting infectious diseases.

The article would be considerably stronger with a comparison to even just one existing approach, e.g. in the mean squared error of predicted case counts. Justification should also be given for the choice of model to compare against, though greater generality might be achieved by comparing against [4], given that they already present quantitative comparisons with approaches the authors of this article are citing (e.g. McGough et al.). It should also be noted that prediction error isn’t everything, and comparisons between run times, complexity of code, and accessibility to a wide range of practitioners can also help to build a compelling argument for operational use.

Minor comments:

5. Some fonts on the figures are too small to read without zooming in significantly, please consider making these fonts larger.

6. The authors mention the possibility of using a regression tree approach in the Discussion section. Are there any significant obstacles to simply passing through all of the inputs to, say, the first network model, into a random forest?

7. Are any of the models proposed able to account for structured variability in the reporting delay, for example the improvement or deterioration in reporting times over a number of years?

8. Are the proposed models applicable in the situation where only a short time series of data exists, e.g. when dealing with daily case counts in the first few weeks of a new pandemic?
