# Supplementary material for: A nowcasting framework for correcting for reporting delays in malaria surveillance
Source: PLoS Comput Biol. 2021 Nov 16;17(11):e1009570. doi: 10.1371/journal.pcbi.1009570 (PMC8659367; doi:10.1371/journal.pcbi.1009570)
Supplement: Attachment — Submitted filename: Response to Reviewers - Guyana nowcasting manuscipt.pdf [file pcbi.1009570.s012.pdf]

Response to reviewers

Reviewer #1: Review uploaded as an attachment. (pasted below)

Summary of review:
The article is a clearly written and distinct contribution to the literature on correcting reporting delays and nowcasting diseases, which is currently receiving considerable attention due to the ongoing pandemic.
The authors have done a great job of motivating the problem of reporting delays through their compelling application to malaria in Guyana. I particularly appreciate the descriptions of specific causes of reporting delays in the introduction.
The authors present two classes of models and their application to the malaria data. Leveraging these models and a more descriptive analysis, the authors discuss new insights into the data, including the spatial distribution of reporting delays, and demonstrate a level of effectiveness as a tool for nowcasting case counts.
However, I do believe there are some weaknesses in the article which should be addressed prior to publication.

We thank the reviewer for this positive assessment of our work.

Major comments:
1. The authors make a potentially misleading claim about other approaches, specifically those designed in the Bayesian framework.
"One limitation of the Bayesian methods is that they do not focus on providing more interpretable measures to guide actionable surveillance efforts, such as direct point estimates for predicted case counts." The cited Bayesian approaches (e.g. McGough et al. and Bastos et al.) are capable of producing *both* point estimates (e.g. posterior median, posterior mode) and measures of uncertainty (e.g. 95% prediction intervals) for the total number of cases. I believe these point estimates are no less valid or direct than point estimates derived by minimising a mean squared error.

Many thanks for pointing this out; we do agree with your sentiments about the equal validity of point estimates and posterior means or other summary estimates . We have removed this statement to avoid expressing any indication that the former is intrinsically favored over the latter.

2. The authors do not address the lack of measures of uncertainty in their approach, particularly relating to the predicted case counts.
Given that all nowcasting predictions from any model will be in some sense "wrong", it is my opinion that measures of uncertainty are important for disease surveillance applications, both to quantify how wrong the predictions might be and also, for example, to inform decision-makers about possible scenarios other than the most likely scenario. Measures of uncertainty are apparently lacking from the authors' approach, though they don't acknowledge this as a potential drawback.
If used in an operational setting over a period of time, one would begin to collect information about the (out-of-sample) prediction errors of nowcasts from these models. Could you derive approximate measures of prediction uncertainty from the distribution of these errors? For example (I am not specifically recommending this solution), in unpublished works, I have previously derived crude intervals from twice

the standard deviation (without any statistical justification) of the last N errors, so that uncertainty is measured in a moving window.

We thank the reviewer for this critical comment about the need to quantify the degree of uncertainty surrounding our point estimates and the possible range of case counts policy-makers can reasonably expect. We have updated our analysis to include moving confidence bounds for all regional point estimates, starting from January 2009 following an approach similar to the one you outlined and that described in [Yang et al., 2015 and Poirier et al., 2021]. This method accommodates the changing nature of reporting trends over time by relying on the errors of the predictions for the two years preceding each month.

3. The authors have missed out a growing literature of Bayesian approaches which combine a model for the total case counts occurring in each time period with a conditional model for the reporting delay. These hierarchical approaches result in a predictive distribution for the total case counts, directly informed by all available partial observations of the number of cases and previously observed case counts. In my opinion, these approaches, particularly those based on the Generalized-Dirichlet-Multinomial framework, represent the current "gold standard" of nowcasting infectious disease. Therefore, I would like to see these discussed alongside the other existing approaches.
Originating article for the GDM framework:
[1] https://doi.org/10.1111/biom.13188
Applications to COVID-19:
[2] https://arxiv.org/pdf/2102.04544.pdf
[3] https://doi.org/10.1101/2020.09.15.20194209
[4] https://arxiv.org/abs/1912.05965

We thank the reviewer for highlighting these additional model applications, which we have now cited in our discussion of existing Bayesian methods that dually consider reporting trends and known case trends. Following your comment, we have also reinforced that these Bayesian methods serve as the existing "gold standard" approach.

4. While the proposed models are apparently novel, without any quantitative comparisons with other approaches it is difficult to place them in the context of existing approaches and therefore determine the value of their contribution to the problem of nowcasting infectious diseases.
The article would be considerably stronger with a comparison to even just one existing approach, e.g. in the mean squared error of predicted case counts. Justification should also be given for the choice of model to compare against, though greater generality might be achieved by comparing against [4], given that they already present quantitative comparisons with approaches the authors of this article are citing (e.g. McGough et al.). It should also be noted that prediction error isn't everything, and comparisons between run times, complexity of code, and accessibility to a wide range of practitioners can also help to build a compelling argument for operational use.

We appreciate this important suggestion. We now compare our models to that of a Bastos et al. model, a Bayesian approach aimed at uncovering partially unknown converged case counts, i.e. the "runoff triangle". [Bastos, 2019], with an application to dengue data in Brazil.

In addition to our comparison with the Bastos et al. model, we have also expanded our discussion of our proposed methods, including an ensemble approach we introduced, to consider additional benefits beyond error rates, notably run times and model transparency.

Minor comments:
5. Some fonts on the figures are too small to read without zooming in significantly, please consider making these fonts larger.

We have increased the size of all text in our figure legends.

6. The authors mention the possibility of using a regression tree approach in the Discussion section. Are there any significant obstacles to simply passing through all of the inputs to, say, the first network model, into a random forest?

We agree that implementing an ensemble regression tree approach could be of interest as an alternative method, but note in the discussion that it is not of primary interest of this study, rather, an avenue for future research, particularly for extensions that consider a wider range of input variables. We also note that introducing an additional and potentially more complex method, which necessitates assumptions about far more hyperparameters, may perhaps undermine the goal of ensuring broad accessibility to decision-makers at the Guyana Vector Control Services and Ministry of Health.

7. Are any of the models proposed able to account for structured variability in the reporting delay, for example the improvement or deterioration in reporting times over a number of years?

Yes. One of the benefits of our proposed method is that, via a moving window training and testing approach, it accounts for any changing trends in the quality of surveillance programs and the extent to which cases are reported each month, which we note in the text.

8. Are the proposed models applicable in the situation where only a short time series of data exists, e.g. when dealing with daily case counts in the first few weeks of a new pandemic?

Our methods are not applicable to emerging outbreak/epidemic settings, given that they require sufficient historical period for training (at least 12 months).

Reviewer #2: general comments:

I believe the presentation of the methodology could greatly benefit from the adition of a figure containing some kind of flow diagram of the entire analysis from the Imputation through to the Nowcasting.

We thank the reviewer for this suggestion. We have now included a flow diagram detailing each of the key steps in the data preparation to model implementation process (Supplementary Flowchart F1)

A basic aspect of malaria epidemiology, is that it may persist on the same patient for a long time with many relapse episodes. Thus when a temporal cluster of cases is observed, it is not necessarily indicative of an outbreak of new infections. Besides, clusters of case reports may also be influenced by

observational biases, for example when health professionals visit a remote village and test the whole community in a short interval of time. The authors must explain how they hope to describe incidence patterns given these confounding factors. Some reported cases may also be a relapse of the same patients.

We agree that these are important considerations regarding traditional malaria epidemiological trends and characteristics of surveillance efforts. A fraction of patients (28%) were in fact indicated to be relapses, a majority of whom were infected by P. vivax, which is consistent with the species' role in recurrent cases. However, the aim of our analysis was to inform more accurate estimates of recorded cases that are not reported to the central office in Georgetown until several weeks to months later, rather than a mechanistic simulation of malaria transmission trends. Thus, the presence of relapse cases does not necessarily hinder our study, a point we now make in the methods. Regarding observational biases, we clarify in the introduction that active surveillance is the primary mode of identification of malaria patients, as they are largely seen, examined, and/or treated at health facilities, with a very small percentage of cases (8%) detected through community outreach programs.

Regarding the Nowcasting models, I think that calling the first regression model an "imputation" is not entirely accurate because the model actually estimates cases which have occurred but not yet been reported at time t. Imputation is when you replace missing data with something else. Even though these revised case counts are used in the subsequent models, this alone is different from a standard data imputation scheme.

While we agree that "imputation" may connote methods used to resolve missing data issues, we note that it is a commonly used term in modeling literature to refer to nowcasting methods which can improve on case count data afflicted by missingness due to reporting delays.

The authors then describe two types of "network" models that also include the previous 12 months of data from all other regions to predict for a given region. Agin here I think that calling a network model is misleading since no particular influence network between the regions is being considered, instead all the regions are included. Moreover, from the text alone, the distinction between the first network model and the second is not easy to grasp, I think the authors should either improve the textual description or add a figure to help tease apart the two models' structures.

We also note that nowcasting models are a commonly used phrase in the nowcasting literature to denote methods which incorporate epidemiology information across geographic areas, even if there is no explicit weighting of all other regions' influence on a current region's converged case trends. For instance, the Lu et al paper we cite employs this "network-based" language in reference to their ARGONet model [Lu et al., 2015]. We have reframed our presentation of the network model in the methods to better characterize the network of malaria activity across regions that is used to inform each region's converged case count.

To address the reviewer's second suggestion, we have now included a supplementary figure (Supplementary Diagram D2) to help readers distinguish between the two network models with respect to model inputs.

In the first paragraph of the Nowcasting section, the authors say that "...The first out-of-sample case count estimate for all regions was produced for January of 2007 using historical information available at the time (training set time period) that consisted of data from the previous 12 months (within 2006)..." And

they also say that "Subsequent estimates were produced by dynamically training the models... as more information became available...". If they continued to train the model on the new data, then none of the estimates produced by the model were trully "out of sample". This statement must be better justified or corrected. A more traditional out-ofsample validation scheme should be presented where the validation set would be separated beforehand and used only to for predicting purposes, once the training is done. There's nothing wrong with a rolling window within the training set.

We do agree that referring to our model estimates as part of an 'out-of-sample' model testing procedure over the entirety of our dataset does not necessarily reflect traditional approaches that fully dissociate training and testing datasets. To this end, we expand our discussion of this validation approach to underscore that this 'out-of-sample' data assimilation process does in fact separate training and testing datasets at each month a prediction is made. For example, starting with January 2007, case counts only known by January 2007 are used as predictors and targets in training, with the identified optimal model then used to predict the yet to be known January 2007 full case count.

Confidence intervals for the the revised case counts should be added to figures 3 and 4.

Many thanks for this comment. We have now included confidence intervals on all figures of model inputs, starting with point estimates from January 2009. Please refer to our methods section for a description of our method of obtaining confidence intervals.

Reviewer #3: The authors have analyzed 13 years of malaria surveillance data from Guyana. They developed methods to "nowcast" cases, meaning that they used the historical data as it would have been available to the program, and they developed methods to accurately predict the actual number of cases that would eventually be reported. The idea is not particularly new, but the study was well-done. The paper was well-written. The idea is important and interesting. I recommend accepting the article, though I would like to make a few suggestions to the authors to be considered (or rejected) as they wish.

We thank the reviewer for this thoughtful assessment of our work.

First, I think it would be good to have a simple table summarizing some very basic things. How many people lived in each district? How many malaria cases were reported from each district in each year? Thus what was the incidence of confirmed malaria cases, measured as incidence per 1,000 population, per year? What was the median delay for each district?

Many thanks for the suggestion to report these key descriptive statistics. We now include a figure (Supplementary Figure S) which reports confirmed cases each year from 2006-2019 and median delays for each region. We chose not to include measures of incidence given that all regions, particularly mining areas, consist of largely mobile populations; as such, annual population size estimates are difficult to measure and fail to capture frequent intra-annual population flux

Second, it's dissatisfying that the models failed to converge for district 9. It looks like it might be the district where the method would be most useful. Is there anything you can do? The supplementary figure S1 makes it look like there was a pretty reasonable pattern in the delay for Region 9, so I'm scratching my

head about why. If the problem is that there were too few cases, then that line in the table I suggested would make it clear if it was a low number problem.

While we agree that nowcasts for region 9 would be particularly beneficial in improving surveillance efforts in the region, none of the models we implemented were well-suited to region 9. This failure to converge is driven by the dearth of monthly data on known cases, which compromises the ability of the models to learn from previous trends in reported cases, irrespective of any regularity of the delay pattern. Following your suggestion, we have reinforced this challenge of data sparsity and its consequences for region 9 in our caption for Supplementary Figure S1. Fortunately, we found that the Bastos et al. [2019] model to which we compared our models was able to provide estimates for region 9, which we note in the text could be a useful alternative for this region in particular.

Third, I note that you have three methods (for districts 1, 7, and 8) that work well. Sometimes having three good methods is worse than having just one, if only because it creates confusion about which one to use. I think it would increase the cool factor of the paper and also probably help programs know what to do if you created an ensemble prediction combining the three methods.

We appreciate this important point and suggestion. We have introduced an ensemble model that considers model estimates from each of the three models in recent months and selects the estimate from the best performing model for the current target month (described in the Methods section).

Fourth, if the dashed red lines and the solid blue lines are same for every district in Figures 3 and 4 (for districts 1,7,and 8), why not consolidate? It would be easier to see the differences in the predictions made by various methods (give each method a different color).

Many thanks for this comment. We attempted to consolidate all model predictions in one figure, but found that the graph became quite packed and that each of the model's distinct confidence bounds could not be distinguished. However, we did opt to include a consolidated figure in the appendix to give readers the option to view all predictions, known, and converged case trends in one figure (Supplementary Figure S6) if they so choose.

I hope these will be considered suggestions by both editors and authors and not cause the authors stress or grief. If what I'm suggesting is out of line, please explain why it's not possible.

Reviewer #4: "A nowcasting framework for correcting for reporting delays in malaria surveillance" by Menkir et al. is a manuscript that proposes a method to anticipate malaria cases in Guiana based on historical data. Methods for delay correction have been applied to HIV, influenza, dengue fever, and more recently in COVID-19. As far as I am concern it is the first time it has been implemented in malaria, where notification delay is an important issue to be dealt with in an endemic region. Malaria endemic countries in general lack good infrastructure in countryside regions leading to large delays, Guiana faces the same issue. The manuscript aims to tackle an important issue in Guiana and the authors deliver a solution which I think it is indeed simple and useful however it lacks a better literature review on nowcasting methods and also a comparison between the proposed methods and the existing ones (or a justification for not doing this comparison), and a better description of the proposed models is needed. On the good side, their proposed model has a good performance both visually and using the rRMSE.

We thank the reviewer for the positive assessment of our work.

Some comments:

On page 2 the authors said "One limitation of the Bayesian methods is that they do not focus on providing more interpretable measures to guide actionable surveillance efforts, such as direct point estimates for predicted case counts." The Bayesian methods described are all based on the chain-ladder method, a relatively well-known tool in actuarial sciences used to calculate incurred but not reported (IBNR) loss estimates (Renshaw, 1989). The models mentioned in the manuscript (Rotejanaprasert t al., 2020; McGough et al., 2020; Bastos et al., 2019). There are both Bayesian and non-Bayesian approaches and I failed to see the described limitation since in all recent recent approaches one can have more than a point estimate of case counts, they all can provide a predictive distribution of the case counts.

Many thanks for this comment and for placing the methods we cite in the introduction in context; we now include the additional references you recommended, including that of Renshaw 1989, to provide this additional context. We also agree with your statement about both categories of methods capable of providing point estimates, whether they be direct point estimates or summary measures (as in the case of the Bayesian approaches), so we have removed the statement making this distinction.

The literature review of nowcasting models in infectious diseases should also include Salmon et al. (2015) and Stoner and Economou (2020), they both provide extensions of the chain-ladder model and apply their proposed methods into infectious diseases data. More recently there are these extensions trying to model COVID-19 deaths (Hawryluk et al., 2020; Seaman et al.; 2020). I am not suggesting an exhausting literature review on methods, but there is some literature on the matter and I expected some comparison to existing methods, or perhaps a better description saying why the existing methods are not suitable in these context,

Thank you for highlighting these additional applications, which we now cite in our introduction. Regarding your crucial point about model comparisons, we now evaluate our model against a Bayesian approach also used to estimate vector-borne case trends (Dengue) in Brazil [Bastos, 2019].

Data description: Could the authors describe the data? How is the delay calculated? On page 5 there is odd notation `~\bar{y}_i(t*)` that "denotes the number of cases occurring in each month t* known by the end of month t". Should that be \widetilde{y}_i(t*)? But where is t in this notation? How can \widetilde{y}_i(t*) at time t-1 be different than \widetilde{y}_i(t*) at time t? I might be confused with the notation here. It would be important to make it clearer.

Many thanks for your comment. We have now included a brief overview of the central database in the methods. We have also reiterated how delays are calculated, i.e. the difference in days from when a patient's smear is examined and when the patient is inputted in the central database, in Supplementary Diagram D1. We have also corrected the notation regarding the model equation you are referring to and clarified any additional variables we did not explicitly define.

About the models: DIM is a baseline model using as covariates the number of cases for the past twelve months observed at time t, in addition to that there is an assumption that these coefficients do not vary in space, since h is not indexed by region. NM-1, the {h} coefficients are not indexed by region, is that correct? I believe they vary by region, i.e. y_i(t) \sim \sum_{j \neq i} \sum_k h_{(j-1)*12 + k} \widetilde{y}_{j}(t-k). This is also valid for model NM-2, which extends model NM-1 by adding N-1 covariates, the predict cases of regions j different than i using model DIM. Is that correct? The notation in equation (1-3) is not clear, another common notation in a modelling approach is the use of tilde y ~ a*x + b*z, but is there probability distribution behind the elastic net penalized model? By using the OLS can I assume a Gaussian distribution is assumed in the background?

Thank you for pointing this omission out; you are correct, the h coefficients are indexed by region; we have now adjusted the model equations for NM1 and NM2 accordingly. We have also added a note that we are indeed assuming a Gaussian distribution.

Uncertainty: The uncertainty of the predictions is ignored. It should have a justification for ignoring it. The authors consider only a predictive point estimate. I assume that their proposed methods allow to estimate intervals for the predictions (nowcasts). Hence I would expect a uncertainty interval for the solid red lines in Figures 3 and 4. For models DIM and NM-1 I assume the intervals are straightforward to obtain, for NM-2 is not so direct because there is some uncertainty on the predictive value used as a covariate that should be propagated.

Many thanks for this critical feedback. We have now included confidence intervals for all nowcasts, which we generated following a moving confidence interval approach outlined in Lu et al. [2019], in which we use information on the root mean square error of the prior two years of predictions to bound monthly point estimates, starting with January 2009.

Code and data sharing: Would R code and data be available? Even if the authors opt to not compare their methods with other nowcasting methods it would be very interesting to evaluate the performance of the elastic net penalized regression for nowcasting.

All our code can be accessed here: https://github.com/goshgondar2018/guyana_nowcasting. Unfortunately, due to our data sharing agreement, we are unable to share the routine diagnosis data.

Minor comments:

Equation 1: The subscripts i on the left hand side and l (?) on the right hand side of the equation need to be describe.

We state what i index denotes in the introduction of the synchronous data imputation model.

Page 6: There is no such version R version 1.2.1335, the version 1.2.2 was released in 2001. I assume 1.2.1335 refers to RStudio version, in order to find out the R version type "version" on R console.

Thank you for noting this. We have corrected this statement.

Refs:

Hawryluk, Iwona, et al. "Gaussian Process Nowcasting: Application to COVID-19 Mortality Reporting." arXiv preprint arXiv:2102.11249 (2021).

Renshaw, A.E. "Chain ladder and interactive modelling (claims reserving and GLIM)." Journal of the Institute of Actuaries (1886-1994) 116.3 (1989): 559-587.

Salmon, M., Schumacher, D., Stark, K. and Höhle, M. (2015) Bayesian outbreak detection in the presence of reporting delays. Biometrical Journal, 57, 1051-1067.

Seaman, Shaun, et al. "Nowcasting CoVID-19 Deaths in England by Age and Region." medRxiv (2020).

Stoner O, Economou T. (2020) Multivariate hierarchical frameworks for modeling delayed reporting in count data, Biometrics, volume 76, no. 3, pages 789-798, DOI:10.1111/biom.13188.
