# Supplementary material for: A nowcasting framework for correcting for reporting delays in malaria surveillance
Source: PLoS Comput Biol. 2021 Nov 16;17(11):e1009570. doi: 10.1371/journal.pcbi.1009570 (PMC8659367; doi:10.1371/journal.pcbi.1009570)
Supplement: Attachment — Submitted filename: Revision 2 Guyana Manuscript - response to reviewers.docx [file pcbi.1009570.s013.docx]

Dear Editor,

We are writing to provide an update to our manuscript, “A nowcasting framework for correcting for reporting delays in malaria surveillance” by Menkir et al., consistent with additional feedback provided by reviewer #4. Please find below the response to the reviewer’s suggestion.

Sincerely yours,

Caroline Buckee, PhD

cbuckee@hsph.harvard.edu

*Response to review*

Reviewer #4: The authors have answered all of my questions, and I am happy with the manuscript. It is well-written and tackles a relevant public health problem not only for Guiana, but for all countries where malaria is endemic and there is a case notification system.

I would like to make a suggestion though. The estimates for both models data imputation model (DIM) and networks models (NM1 and NM2) return negative estimates for the number of cases (Figures 3 and 4). So I wonder if use a log-transformation in the modelling approach and then transform back to the original scale for the estimates would improve the results. It would certainly avoid the negative estimates, but I am not sure if it would somehow mess up with the uncertainty. Since it is a cheap model to run, it may be worth testing.

We thank the reviewer for this important suggestion. After instituting the proposed solution to log transform the target case counts to mitigate the negative predictions observed across all models, we didn’t observe universal improvements across regions. While it did ensure all predicted case counts were positive, the logged data imputation model for region 1 performed considerably worse overall, as did its two network models. The opposite was true for the region 7 and 8 models, but any improvements in model performance were modest. Due to the mixed results in model performance across regions, we preserved our original method, but now comment in our discussion that we explored this alternative.
